# Red Blood Cell Distribution Width Is a Predictive Factor of Anthracycline-Induced Cardiotoxicity

**DOI:** 10.3389/fcvm.2020.594685

**Published:** 2020-10-30

**Authors:** Daiki Yaegashi, Masayoshi Oikawa, Tetsuro Yokokawa, Tomofumi Misaka, Atsushi Kobayashi, Takashi Kaneshiro, Akiomi Yoshihisa, Kazuhiko Nakazato, Takafumi Ishida, Yasuchika Takeishi

**Affiliations:** Department of Cardiovascular Medicine, Fukushima Medical University, Fukushima, Japan

**Keywords:** cardio-oncology, anthracycline, red blood cell distribution width, cancer therapeutics-related cardiac dysfunction, heart failure 2

## Abstract

**Background:** Red blood cell distribution width (RDW) is associated with prognosis in widespread cardiovascular fields, but little is known about relationship with the onset of cancer therapeutics-related cardiac dysfunction (CTRCD).

**Objectives:** The purpose of this study was to assess whether RDW could predict the onset of CTRCD by anthracycline.

**Methods:** Consequential 202 cancer patients planed for anthracycline treatment were enrolled and followed up for 12 months. The patients were divided into 2 groups based on the median value of baseline RDW before chemotherapy [low RDW group, *n* = 98, 13.0 [12.6–13.2]; high RDW group, *n* = 104, 14.9 [13.9–17.0]]. Cardiac function was assessed serially by echocardiography at baseline (before chemotherapy), as well as at 3, 6, and 12 months after chemotherapy with anthracycline.

**Results:** Baseline left ventricular end systolic volume index and ejection fraction (EF) were similar between two groups. After chemotherapy, EF decreased at 3- and 6-month in the high RDW group [baseline, 64.5% [61.9–68.9%]; 3-month, 62.6% [60.4–66.9%]; 6-month, 63.9% [60.0–67.9%]; 12-month, 64.7% [60.8–67.0%], *P* = 0.04], but no change was observed in low RDW group. The occurrence of CTRCD was higher in high RDW group than in low RDW group (11.5 vs. 2.0%, *P* = 0.008). When we set the cut-off value of RDW at 13.8, sensitivity and specificity to predict CTRCD were 84.6 and 62.0%, respectively. Multivariable logistic regression analysis revealed that baseline RDW value was an independent predictor of the development of CTRCD [odds ratio 1.390, 95% CI [1.09–1.78], *P* = 0.008]. The value of net reclassification index (NRI) and integrated discrimination improvement (IDI) for detecting CTRCD reached statistical significance when baseline RDW value was added to the regression model including known risk factors such as cumulative anthracycline dose, EF, albumin, and the presence of hypertension; 0.9252 (95%CI 0.4103–1.4402, *P* < 0.001) for NRI and 0.1125 (95%CI 0.0078–0.2171, *P* = 0.035) for IDI.

**Conclusions:** Baseline RDW is a novel parameter to predict anthracycline-induced CTRCD.

## Introduction

Anthracycline-containing chemotherapy is highly effective and widely used in cancer treatments. However, anthracycline-induced cardiotoxicity is associated with a poor prognosis in cancer survivors, and the frequency of onset depends on risk factors including cumulative dose of anthracycline, elderly or pediatric population, concomitant or previous radiation therapy, pre-existing cardiovascular diseases, and concomitant use of human epidermal growth factor receptor 2 (HER2) inhibitors (1). Several hypotheses have been proposed for the mechanisms of anthracycline-induced cardiotoxicity, such as oxidative stress, iron accumulation in myocardial cells, mitochondrial dysfunction, and topoisomerase 2β dysfunction (2–5). Most anthracycline-induced cardiotoxicity occurs within the first year of chemotherapy, and early detection and treatment may recover cardiac function (6). Therefore, assessment of cardiac function should be performed to detect early phase of cardiotoxicity, and accurate predictors are required to identify patients predisposed to anthracycline-induced cardiotoxicity.

Red blood cell distribution width (RDW) is a simple and rapid measurement of the heterogeneity of erythrocyte volume. Growing body of evidence suggests that high RDW is strongly associated with poor prognosis in widespread cardiovascular diseases such as acute coronary syndrome, heart failure, and pulmonary hypertension (7–9). However, the association between anthracycline-induced cardiotoxicity and RDW have not been rigorously examined. The purpose of the present study was to assess whether baseline RDW could predict the onset of cancer therapeutics-related cardiac dysfunction (CTRCD) by anthracycline-containing chemotherapy.

## Methods

### Study Subjects and Protocol

**Figure 1:**
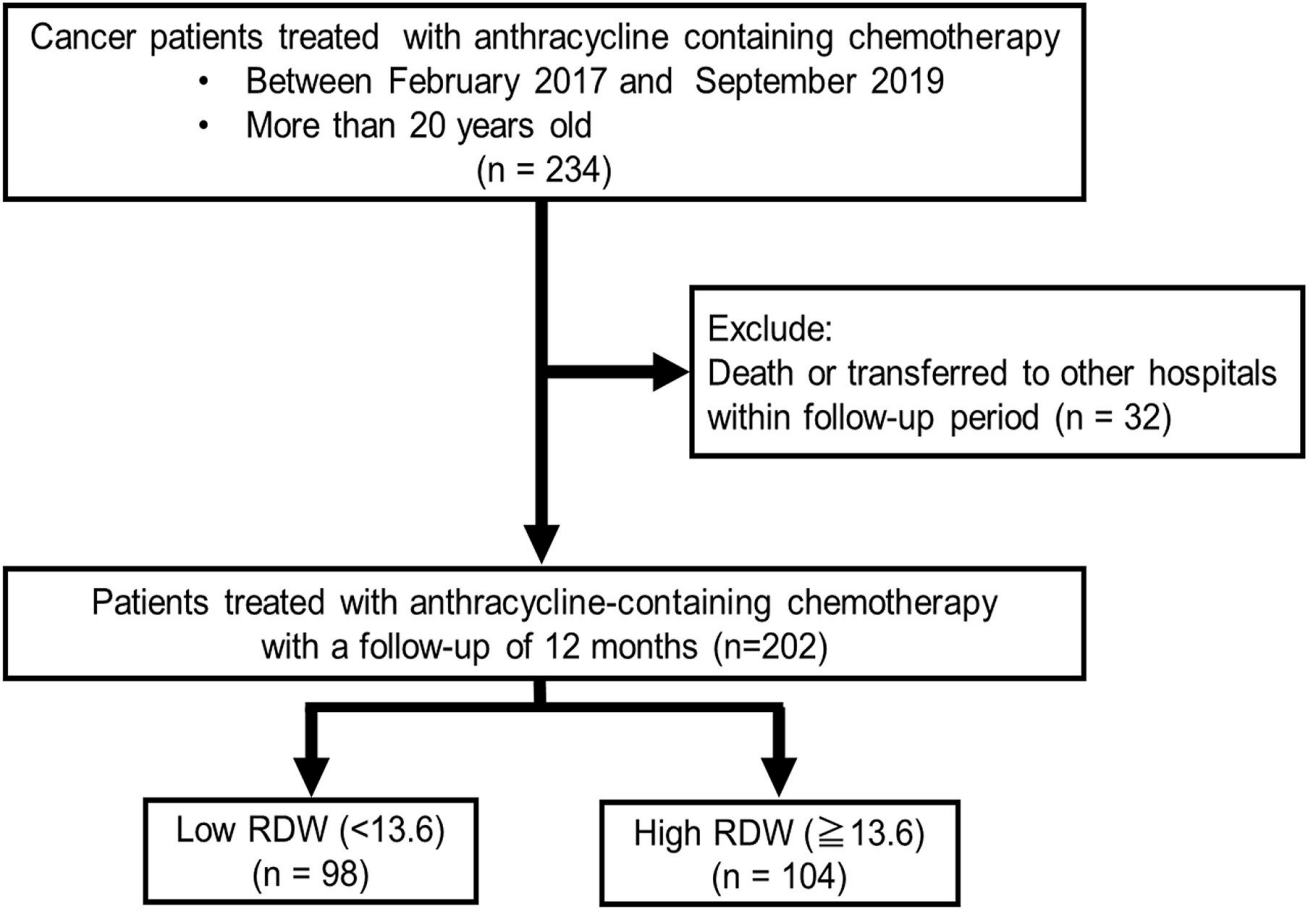
Patient cohort selection.

We enrolled 234 consecutive cancer patients, planned for initial anthracycline-containing chemotherapy at Fukushima Medical University hospital from February 2017 to September 2019 (Figure 1). Patients were excluded if they were died or transferred to other hospitals within 12 months follow-up period (*n* = 32). Remaining 202 patients were divided into 2 groups based on the median value of RDW (13.6) before chemotherapy: low RDW group, *n* = 98, 13.0 [12.6–13.2] and high RDW group, *n* = 104, 14.9 [13.9–17.0].

Hypertension was defined as a history of use of antihypertensive drug or systolic blood pressure of ≥140 mmHg, and/or diastolic blood pressure ≥90 mmHg. Diabetes was defined as a recent use of insulin treatment or hypoglycemic drug, or hemoglobin A1c ≥6.5%. Dyslipidemia was defined as a history of use of cholesterol-lowering drugs, or triglyceride was ≥150 mg/dl, low density lipoprotein cholesterol was ≥140 mg/dl, and/or high-density lipoprotein cholesterol was ≤40 mg/dl. Cumulative dose of anthracycline was expressed as a doxorubicin equivalent (1). HER 2 inhibitor included trastuzumab and pertuzumab. Radiation therapy was defined as irradiation to the mediastinum. Transthoracic echocardiography and blood sampling test were performed at baseline, as well as at 3, 6, and 12 months after administration of anthracyclines. All procedures used in this research were approved by the Ethical Committee of Fukushima Medical University Hospital.

### Echocardiography

Transthoracic echocardiography was performed by a trained sonographer, and images were checked by another trained sonographer and an echo-cardiologist. We measured cardiac function using EPIQ 7G (Philips Healthtech, Best, Netherland). EF was calculated using the modified Simpson's method according to the guideline from the American Society of Echocardiography and the European Association of Cardiovascular Imaging (10). The left ventricular (LV) mass was calculated using the following formula:

$$\begin{array}{l} \text{Left}\;\text{ventricular}\;\left(\text{LV}\right)\;\text{mass}\\ =0.8\times [1.04\times \text{\{(}\text{LV}\;\text{diastolic}\;\text{diameter}+\text{interventricular}\\ {\text{septum}\;\text{wall}\;\text{thicness}+\text{LV}\;\text{posterior}\;\text{wall}\;\text{thicness})}^{3}\\ -{\left(\text{LV}\;\text{diastolic}\;\text{diameter}\right)}^{3}\}]+0.6\text{g}[10]. \end{array}$$

CTRCD was defined as a decrease in EF more than 10% points, to a value <53% (11). LV diastolic dimension index, LV systolic dimension index, LV end-diastolic volume index, LV end-systolic volume index, and left atrial volume index (LAVI) were measured using B-mode ultrasound.

### Blood Sampling

Blood sampling was performed in a stable condition after an overnight fast. High sensitivity cardiac troponin I (TnI) was measured using an assay based on Luminescent Oxygen Channeling Immunoassay technology, and run on a Dimension EXL integrated chemistry system (Siemens Healthcare Diagnostics, Deerfield, IL, USA). B-type natriuretic peptide (BNP) levels were measured using a specific immunoradiometric assay (Shionoria BNP kit, Shionogi, Osaka, Japan). RDW was measured using a DxH800 (Beckman Coulter, Inc., Fullerton, CA, USA).

### Statistical Analysis

All statistical analyzes were performed using SPSS version 26 (IBM, Armonk, New York, USA) or R software packages version 3.6.3 (R core team 2020, Vienna, Austria). We used the Shapiro-Wilk test to discriminate which variables were normally or not normally distributed. Normally distributed variables were shown as mean ± standard deviation. Non-normally distributed variables were indicated by median with interquartile range. Category variables were shown in numbers and percentage. Student's *t-*test was used for variables following a normal distribution, the Mann-Whitney *U-*test was used for variables of the non-normal distribution, and the χ-square test was used for categorical variables. The time course of EF (baseline, 3-, 6-, and 12-month after the administration of anthracyclines) was evaluated using the Friedman test.

Logistic regression analysis was performed to identify variables relating to the occurrence of CTRCD. We selected variables as follows: age, sex, hypertension, dyslipidemia, diabetes mellitus, current or past smoker, cumulative anthracycline dose, use of HER2 inhibitor, BNP, albumin, LV mass index, LV end-diastolic volume index, LV end-systolic volume index, EF, LAVI, and RDW. Akaike Information Criteria was used for selecting variables for multivariate analysis. Receiver operator characteristic curve (ROC) analysis was performed to determine the optimal cut-off value of RDW for predicting the occurrence of CTRCD. The improvement by adding RDW to discrimination and net reclassification of risks was assessed by the estimation of both integrated discrimination improvement (IDI) and net reclassification improvement (NRI). The *P*-value of 0.05 or less was defined as significant.

## Results

Table 1 showed patient characteristics at the baseline of chemotherapy. The median value of RDW of all patients before chemotherapy was 13.6, and patients were divided into 2 groups based on the median value. Of 202 patients, 104 patients were grouped as high RDW group (13.6 and above), and 98 patients were grouped as low RDW group (<13.6). There were no statistical differences in age, sex, coronary risk factors, cumulative anthracycline dose, the usage of HER2 inhibitor, and radiation therapy between the two groups. High RDW group included lower rate of breast cancer (60.2 vs. 43.2%, *P* = 0.016). Laboratory data showed that hemoglobin and mean corpuscular volume were lower in high RDW group, but BNP and TnI levels at baseline were similar between the two groups. The usage of angiotensin-converting enzyme (ACE) inhibitors, angiotensin II receptor blockers (ARB), β-blockers were similar between the two groups. Echocardiographic data demonstrated that LVEDVI, LVMI, and EF at baseline were similar between the two groups. Laboratory data showed similar liver and renal function.

**Table 1:** Baseline clinical characteristics of patients with high or low RDW.

	**Low RDW (*n =* 98)**	**High RDW (*n =* 104)**	***P*-value**
Age, years	58.0 (47.5–66.0)	54.0 (45.0–65.0)	0.396
Female, n (%)	81 (82.6)	81 (77.8)	0.395
Body mass index, kg/m^2^	22.9 ± 3.7	23.7 ± 4.5	0.161
**Cardiovascular risk factor**			
Hypertension, n (%)	21 (21.4)	26 (25.0)	0.426
Dyslipidemia, n (%)	34 (34.6)	23 (22.1)	0.101
Diabetes mellitus, n (%)	10 (10.2)	12 (11.5)	0.669
Current or past smokers, n (%)	33 (33.6)	36 (34.6)	0.849
Family history of CAD, n (%)	9 (9.1)	14 (13.4)	0.270
CKD (eGFR <60 ml/min/1.73 m^2^), n (%)	15 (15.3)	15 (14.4)	0.858
**Pre-treatment cardiovascular medications**			
Aspirin, n (%)	2 (2.0)	3 (2.8)	0.528
Statin, n (%)	9 (9.1)	10 (9.6)	0.916
β-blocker, n (%)	1 (1.0)	2 (1.9)	0.522
ACE inhibitor and/or ARB, n (%)	18 (18.3)	12 (11.5)	0.173
**Anti-cancer treatment**			
Cumulative anthracycline dose, mg/m^2^	200 (180–240)	180 (150–250)	0.203
HER2 inhibitor, n (%)	15 (15.3)	10 (9.6)	0.220
Radiation therapy, n (%)	7 (7.1)	6 (5.7)	0.774
**Oncological disease**			
Breast cancer, n (%)	59 (60.2)	45 (43.2)	0.016
Hematological tumor, n (%)	20 (20.4)	33 (31.7)	0.068
Gynecologic tumor, n (%)	10 (10.2)	14 (13.4)	0.475
Osteosarcoma, n (%)	8 (8.1)	9 (8.6)	0.900
Other solid tumor, n (%)	1 (1.0)	3 (2.8)	0.333
**Laboratory data**			
BNP, pg/ml	11.2 (6.6–19.8)	12.4 (5.9–22.7)	0.665
Troponin I, ng/ml	0.017 (0.017–0.017)	0.017 (0.017–0.017)	0.776
Total protein, g/dl	7.10 (6.8–7.5)	7.0 (6.7–7.4)	0.187
Albumin, g/dl	4.2 (3.9–4.5)	4.0 (3.8–4.3)	0.003
AST, U/L	18 (15–21)	19 (15–24)	0.788
ALT, U/L	15 (12–21)	14 (10–25)	0.560
LDH, U/L	181 (153–215)	197 (165–244)	0.060
BUN, mg/dl	13.0 (10.7–15.0)	13.0 (10.0–15.0)	0.509
Creatinine, mg/dl	0.65 (0.57–0.76)	0.66 (0.57–0.78)	0.588
eGFR, ml/min/1.73 m^2^	77.0 (63.7–89.2)	72.0 (65.0–89.0)	0.705
Total cholesterol, mg/dl	201.9 ± 36.9	191.5 ± 42.6	0.099
Triglyceride, mg/dl	106 (68.2–166.7)	107.5 (79.7–227.5)	0.979
High density lipoprotein cholesterol, mg/dl	54.9 ± 15.0	53.1 ± 15.5	0.458
Low density lipoprotein cholesterol, mg/dl	120.7 ± 30.9	113.2 ± 36.4	0.157
Hemoglobin A1c, %	5.6 (5.4–5.8)	5.7 (5.5–6.1)	0.185
C-reactive protein, mg/dl	0.11 (0.04–0.38)	0.16 (0.07–0.70)	0.024
Uric acid, mg/dl	4.3 (3.4–5.3)	4.7 (3.6–5.3)	0.876
D dimer, μg/ml	0.5 (0.5–0.925)	0.9 (0.5–2.3)	<0.001
White blood cell, μl	5,550 (4,450–6,800)	5,500 (4,200–7,200)	0.958
Hemoglobin, g/dl	13.1 (12.4–13.8)	11.8 (10.5–13.2)	<0.001
MCV, fl	93.0 ± 4.2	89.2 ± 8.9	<0.001
PLT, × 103/μl	252.5 ± 72.8	246.6 ± 96.1	0.624
RDW	13.0 (12.6–13.2)	14.9 (13.9–17.0)	<0.001
**Echocardiographic data**			
LVDdI, mm/m^2^	26.6 (24.5–29.2)	26.3 (24.5–28.5)	0.604
LVDsI, mm/m^2^	16.2 ± 2.4	16.4 ± 2.8	0.680
LVMI, g/m^2^	67.8 (58.7–82.1)	71.3 (61.8–84.9)	0.076
LVEDVI, ml/m^2^	47.0 ± 13.6	46.6 ± 18.6	0.844
LVESVI, ml/m^2^	16.1 (13.4–20.9)	16.2 (12.4–20.1)	0.668
EF, %	64.1 ± 4.9	65.1 ± 5.2	0.181
LAVI, ml/m^2^	22.8 (17.1–28.4)	22.9 (17.3–31.5)	0.333
E/A	0.97 (0.75–0.20)	1.01 (0.76–1.19)	0.710
Mitral regurgitation (mild and above) n, (%)	20 (20.2)	17 (16.5)	0.793
Aortic regurgitation (mild and above) n, (%)	7 (7.1)	8 (7.8)	0.850
Aortic stenosis (mild and above) n, (%)	0	2 (1.9)	0.259
TR-PG, mmHg	19.3 (16.2–22.3)	19.0 (15.0–23.0)	0.463
RVD, mm	27.2 ± 6.3	28.7 ± 5.6	0.098

*Values are indicated by mean ± SD, median with interquartile range or n (%). Monoclonal antibodies include HER2 inhibitor. Radiation therapy includes only irradiation to the mediastinum. Hemato-oncologic disease include Hodgkin disease, Non-Hodgkin disease, acute myeloid leukemia, acute lymphocytic leukemia, and chronic lymphocytic leukemia. Gynecologic tumor include uterine cancer or sarcoma and ovarian cancer. Other solid tumors include thymoma, soft tissue tumor, teratoma*.

*ACE, angiotensin-converting enzyme; ARB, angiotensin II receptor blocker; BNP, B-type natriuretic peptide; CAD, coronary artery disease; CKD, chronic kidney disease; eGFR, estimated glomerular filtration rate; GLS, global longitudinal strain; HER2, human epidermal growth factor receptor 2; LAVI, left atrial volume index; LV, left ventricular; LVDdI, LV diastolic dimension index; LVDsI, LV systolic dimension index; LVEDVI, LV end-diastolic volume index; EF, ejection fraction; LVESVI, LV end-systolic volume index; LVMI, LV mass index; MCV, mean corpuscular volume; RVD, right ventricular dimension; TR-PG, tricuspid pressure gradient*.

**Figure 2:**
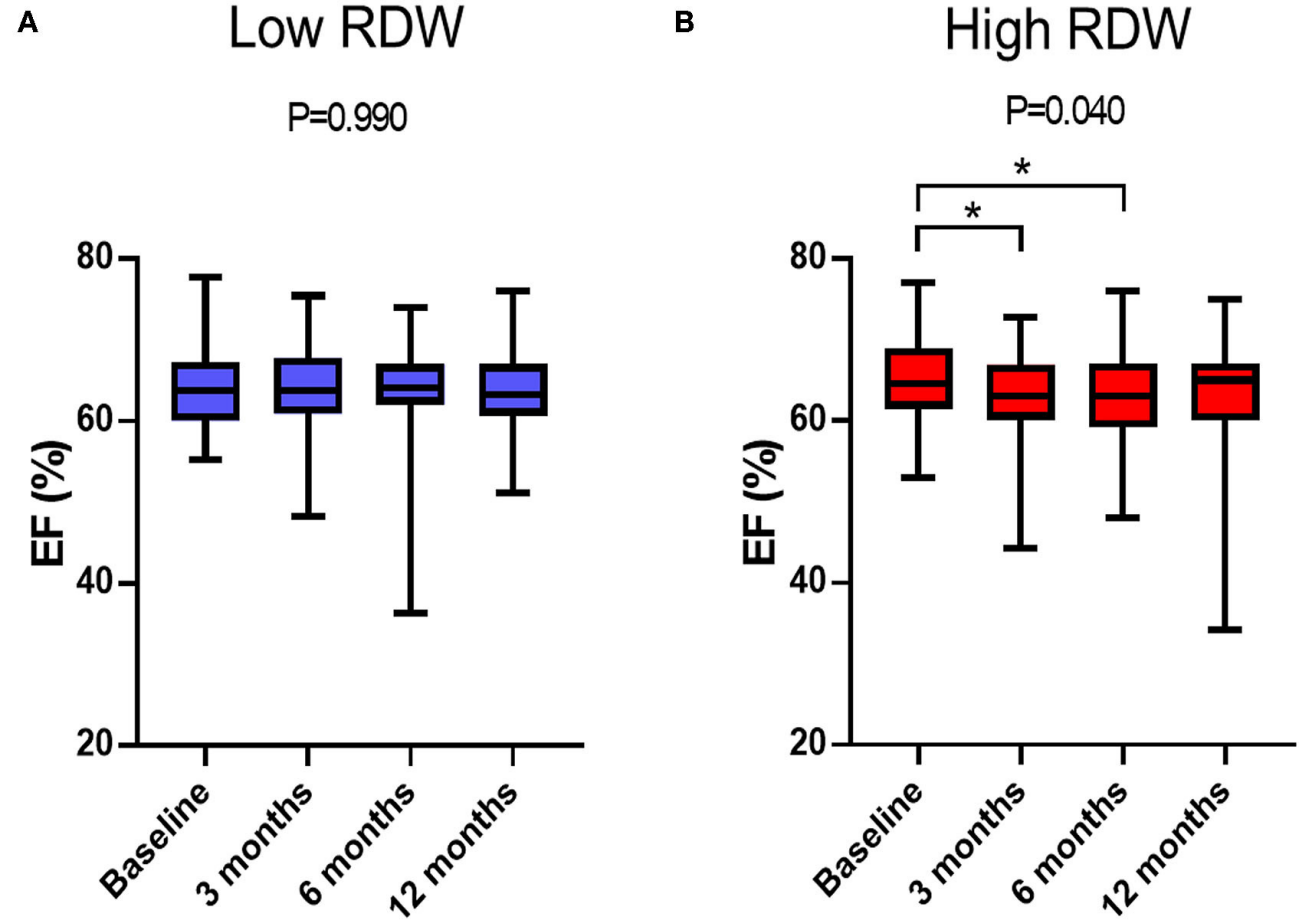
Time-dependent changes in EF after anthracycline treatment. **(A)** Changes in EF in low RDW group. **(B)** Changes in EF in high RDW group. *P < 0.05.

**Figure 3:**
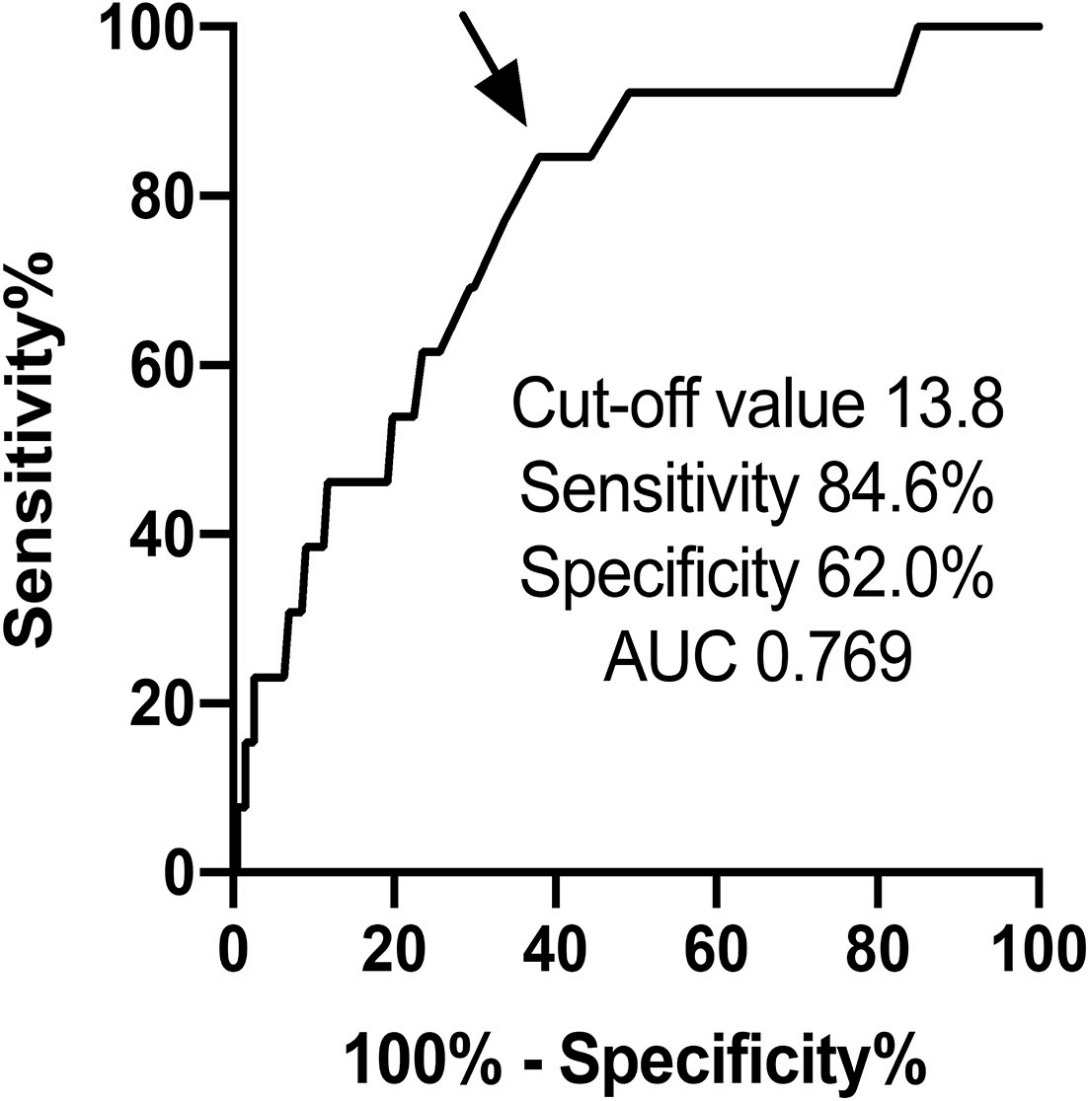
ROC curve analysis of RDW predicting the occurrence of CTRCD by anthracycline.

Time-dependent changes in EF are displayed in Figure 2. EF decreased at 3- and 6-month after chemotherapy in high RDW group [baseline, 64.5% [61.9–68.9%]; 3-month, 62.6% [60.4–66.9%]; 6-month, 63.9% [60.0–67.9%]; 12-month, 64.7% [60.8–67.0%], *P* = 0.04, Figure 2B], but no change was observed in low RDW group (Figure 2A). The occurrence of CTRCD during the 12-month follow-up period was higher in high RDW group than in low RDW group [*n* = 12 [11.5%] vs. *n* = 2 [2.0%] *P* = 0.008]. When we set the cut-off value of RDW at 13.8 from the ROC analysis, sensitivity, specificity, and area under the curve to predict CTRCD were 84.6, 62.0, and 0.769%, respectively, as shown in Figure 3. Multivariable logistic regression analysis revealed that RDW at baseline was an independent predictor of the development of CTRCD [odds ratio 1.390, 95% CI [1.09–1.78], *P* = 0.008; Table 2].

**Table 2:** Parameters associated with the occurrence of CTRCD.

	**Univariable**		Multivariable^*^	
	**Odds Ratio (95% CI)**	***P-*value**	**Odds Ratio (95% CI)**	***P-*value**
Age, per 1-year increase	0.986 (0.950–1.023)	0.455		
Female	0.898 (0.239–0.384)	0.874		
Hypertension	0.511 (0.110–2.371)	0.391	0.264 (0.03–1.78)	0.171
Dyslipidemia	0.186 (0.024–1.446)	0.110		
Diabetes mellitus	0.586 (0.073–4.711)	0.615		
Current or past smoker	0.299 (0.065–1.374)	0.121		
Cumulative anthracycline dose, mg/m^2^ per 1.0 increase	1.010 (1.004–1.016)	0.001	1.010 (1.00–1.02)	<0.001
HER2 inhibitor	0.526 (0.066–4.201)	0.544		
Radiation therapy	1.077 (0.130–8.941)	0.945		
BNP, pg/ml per 1.0 increase	1.000 (0.976–1.025)	0.998		
Albumin, per 1.0 increase	0.469 (0.171–1.286)	0.141	0.367 (0.09–1.35)	0.131
LVMI, g/m^2^ per 1.0 increase	0.998 (0.970–1.027)	0.876		
LVEDVI, ml/m^2^ per 1.0 increase	0.994 (0.959–1.024)	0.574		
LVESVI, ml/m^2^ per 1.0 increase	1.072 (0.970–1.178)	0.149		
EF, per 1.0 increase	0.926 (0.826–1.038)	0.186	0.868 (0.75–1.01)	0.058
LAVI, ml/m^2^ per 1.0 increase	0.999 (0.967–1.031)	0.824		
RDW, per 1.0 increase	1.378 (1.126–1.687)	0.002	1.390 (1.09–1.78)	0.008

* *adjusted for hypertension, cumulative anthracycline dose, albumin, ejection fraction, and RDW categorical variables*.

*CI, Confidence interval; CTRCD, cancer therapeutics-related myocardial disorder; RDW, red blood cell distribution width*.

To assess the importance of adding RDW more precisely, NRI, and IDI were calculated using the variables with or without RDW. The value of NRI and IDI for detecting CTRCD reached statistical significance when baseline RDW value was added to the model including cumulative dose of anthracycline, EF, albumin and hypertension; 0.9252 (95%CI 0.4103–1.4402, *P* < 0.001) for NRI and 0.1125 (95%CI 0.0078–0.2171, *P* = 0.035) for IDI.

## Discussion

In the present study, we revealed the clinical features of RDW in patients treated with anthracycline. First, EF was temporary decreased in high RDW patients. Second, the occurrence of anthracycline-induced CTRCD was significantly higher in high RDW patients. Third, high RDW at baseline was an independent predictor of the development of CTRCD.

Oxidative stress, iron accumulation in myocardial cells, mitochondrial dysfunction, and topoisomerase 2β dysfunction have been proposed as major mechanisms of anthracycline-induced cardiotoxicity (2–5). The excess production of free radicals by the quinone group from aglycons of anthracycline induces oxidation of protein, nucleic acid, and lipid, leading to cellular damage (4). It has been reported that elevation of RDW is also associated with oxidative stress as well as aging, inflammation, malnutrition, and renal dysfunction (12, 13). Among those factors, oxidative stress induces bone marrow dysfunction, leading to abnormal heme synthesis, and hemoglobin production that increased in RDW. In the present study, the patients with high RDW at baseline demonstrated time-dependent decrease in EF and higher occurrence of CTRCD. These findings raise the possibility that the patients with high RDW already had been exposed to increased oxidative stress before chemotherapy, and were more vulnerable to additional oxidative stress by anthracycline, resulting in the development of CTRCD.

Although underlying mechanisms of interaction between RDW and CTRCD remains to be elucidated, to the best of our knowledge, this is the first report to demonstrate the association between RDW and CTRCD.

The utility of RDW has been reported in not only cardiovascular disease, but also cancer prognosis. High RDW are associated with unfavorable survival in many types of cancers including breast cancer (14), hematological tumor (15), gynecological cancer (16, 17), and osteosarcoma (18). Although there is no clear explanation of the relationship between RDW and cancer prognosis, RDW can be a promising biomarker for assessment of the risk of CTRCD and cancer prognosis. Cardinale et al. (6) reported that 78% patients with anthracycline-induced cardiotoxicity showed a full or partial recovery with heart failure therapy. In this cohort, one patient presented <40% of ejection fraction at 6-month follow-up. He was administered β-blockers and ACE inhibitors soon after the onset of CTRCD. Then, his ejection fraction recovered to over 50% at 12-month follow-up. We speculate that early initiation of cardioprotective therapy induced a recovery of cardiac function. Thus, early detection and treatments of CTRCD are essential to minimize cardiac damage induced by anthracyclines. TnI and BNP are widely used surrogate biomarkers to detect CTRCD, but the elevation occurs after starting anthracycline chemotherapy (19). Compared to them, RDW can predict the development of CTRCD before chemotherapy. By assessing RDW before chemotherapy, precise follow-up management can be scheduled, and indication of prophylaxis treatment can be discussed. To date, several clinical studies have demonstrated that angiotensin-converting enzyme and beta-adrenergic receptor blockers prevent cardiac dysfunction after anthracycline chemotherapy (20). According to previous reports of anti-oxidative effects of carvedilol and ACE inhibitor (21), the efficacy of prophylaxis may be expected to the patients with high RDW.

In the present study, the calculation of NRI, and IDI demonstrated the significance of RDW for the prediction of CTRCD. The utility of RDW should be considered when managing cancer patients treated with anthracycline-containing chemotherapy.

## Limitation

This study was performed using a relatively small number of patients and short follow-up period by a single center. Longer follow-up and larger population data were needed to confirm the importance of RDW to the development of CTRCD and cardiovascular prognosis. According to 2016 ESC position paper (1), 3 dimensional-based LVEF have advantages to assess cardiac function compared to Simpson's method, and global longitudinal strain reveals subtle changes in left ventricular function, and thus detects myocardial damage in the early stage before reduces in left ventricular ejection fraction (22). Further studies using 3 dimentional echocardiography and global longitudinal strain are desirable in the future.

## Conclusion

Baseline RDW identifies high risk patients with anthracycline-induced CTRCD.

## Data Availability Statement

The raw data supporting the conclusions of this article will be made available by the authors, without undue reservation.

## Ethics Statement

The studies involving human participants were reviewed and approved by Ethical Committee of Fukushima Medical University Hospital. The patients/participants provided their written informed consent to participate in this study.

## Author Contributions

MO conceived of the presented idea. MO and DY developed the theory and performed the computations. TY, TM, AK, TK, AY, and KN verified the analytical methods. YT and TI supervised the findings of this work. All authors discussed the results and contributed to the final manuscript.

## Conflict of Interest

The authors declare that the research was conducted in the absence of any commercial or financial relationships that could be construed as a potential conflict of interest.
